# Supplementary material for: Modeling glioblastoma heterogeneity as a dynamic network of cell states
Source: Mol Syst Biol. 2021 Sep 16;17(9):e10105. doi: 10.15252/msb.202010105 (PMC8444284; doi:10.15252/msb.202010105)
Supplement: Supplementary file 6 — Source Data for Figure 5 [file MSB-17-e10105-s004.zip › Figure5A_sourcedata/GSEA_3017/nosigdb_stateD.GseaPreranked.1622795010397/RICHARDS_NOWAKOWSKI_SCIENCE_2017_IPC_NEN1_UPREG.html]

Details for gene set RICHARDS\_NOWAKOWSKI\_SCIENCE\_2017\_IPC\_NEN1\_UPREG[GSEA]

|  || Dataset | state23017 |
| Phenotype | NoPhenotypeAvailable |
| Upregulated in class | na\_pos |
| GeneSet | RICHARDS\_NOWAKOWSKI\_SCIENCE\_2017\_IPC\_NEN1\_UPREG |
| Enrichment Score (ES) | 0.13139138 |
| Normalized Enrichment Score (NES) | 1.1897614 |
| Nominal p-value | 0.13513513 |
| FDR q-value | 0.37806937 |
| FWER p-Value | 0.884 |
Table: GSEA Results Summary

  

Fig 1: Enrichment plot: RICHARDS\_NOWAKOWSKI\_SCIENCE\_2017\_IPC\_NEN1\_UPREG      
 Profile of the Running ES Score & Positions of GeneSet Members on the Rank Ordered List

  

| PROBE | GENE SYMBOL | GENE\_TITLE | RANK IN GENE LIST | RANK METRIC SCORE | RUNNING ES | CORE ENRICHMENT || 1 | SOX11 |  |  | 6 | 0.578 | 0.0249 | Yes |
| 2 | DANCR |  |  | 18 | 0.461 | 0.0399 | Yes |
| 3 | MEX3A |  |  | 30 | 0.402 | 0.0518 | Yes |
| 4 | PHLDA1 |  |  | 59 | 0.336 | 0.0469 | Yes |
| 5 | HES6 |  |  | 60 | 0.336 | 0.0642 | Yes |
| 6 | ZEB1 |  |  | 84 | 0.303 | 0.0616 | Yes |
| 7 | SOX4 |  |  | 91 | 0.298 | 0.0721 | Yes |
| 8 | MARCKS |  |  | 96 | 0.295 | 0.0841 | Yes |
| 9 | RBM3 |  |  | 104 | 0.289 | 0.0934 | Yes |
| 10 | FLRT3 |  |  | 106 | 0.289 | 0.1074 | Yes |
| 11 | CHD7 |  |  | 116 | 0.278 | 0.1146 | Yes |
| 12 | TMEM158 |  |  | 120 | 0.275 | 0.1263 | Yes |
| 13 | MMP16 |  |  | 132 | 0.269 | 0.1314 | Yes |
| 14 | RPL8 |  |  | 155 | 0.254 | 0.1270 | No |
| 15 | CELSR2 |  |  | 220 | -0.255 | 0.0895 | No |
| 16 | NF1 |  |  | 254 | -0.260 | 0.0768 | No |
| 17 | TAGLN3 |  |  | 257 | -0.260 | 0.0885 | No |
| 18 | MAPK10 |  |  | 272 | -0.261 | 0.0908 | No |
| 19 | NR2F1 |  |  | 299 | -0.264 | 0.0838 | No |
| 20 | SORBS2 |  |  | 318 | -0.265 | 0.0832 | No |
| 21 | PAG1 |  |  | 330 | -0.267 | 0.0882 | No |
| 22 | MAP6 |  |  | 369 | -0.270 | 0.0720 | No |
| 23 | TMTC2 |  |  | 399 | -0.273 | 0.0631 | No |
| 24 | CPE |  |  | 482 | -0.283 | 0.0128 | No |
| 25 | NREP |  |  | 509 | -0.286 | 0.0069 | No |
| 26 | TSPAN5 |  |  | 513 | -0.287 | 0.0192 | No |
| 27 | SNCAIP |  |  | 540 | -0.290 | 0.0135 | No |
| 28 | HDAC9 |  |  | 548 | -0.291 | 0.0229 | No |
| 29 | LZTS1 |  |  | 564 | -0.293 | 0.0261 | No |
| 30 | PPP2R2B |  |  | 589 | -0.297 | 0.0223 | No |
| 31 | AUTS2 |  |  | 590 | -0.297 | 0.0376 | No |
| 32 | MEIS2 |  |  | 594 | -0.298 | 0.0505 | No |
| 33 | NEDD4L |  |  | 595 | -0.299 | 0.0658 | No |
| 34 | PAX6 |  |  | 629 | -0.307 | 0.0555 | No |
| 35 | ENC1 |  |  | 643 | -0.309 | 0.0610 | No |
| 36 | BICD1 |  |  | 647 | -0.310 | 0.0746 | No |
| 37 | ZFHX4 |  |  | 656 | -0.311 | 0.0842 | No |
| 38 | TMSB4X |  |  | 742 | -0.329 | 0.0339 | No |
| 39 | SOGA1 |  |  | 757 | -0.333 | 0.0399 | No |
| 40 | TRIM2 |  |  | 759 | -0.333 | 0.0562 | No |
| 41 | SOX5 |  |  | 774 | -0.337 | 0.0624 | No |
| 42 | NFIC |  |  | 781 | -0.339 | 0.0750 | No |
| 43 | NKAIN3 |  |  | 828 | -0.351 | 0.0567 | No |
| 44 | RND3 |  |  | 853 | -0.358 | 0.0561 | No |
| 45 | COTL1 |  |  | 907 | -0.376 | 0.0335 | No |
| 46 | CCSAP |  |  | 929 | -0.386 | 0.0367 | No |
| 47 | MACF1 |  |  | 940 | -0.390 | 0.0488 | No |
| 48 | GLI3 |  |  | 1016 | -0.424 | 0.0112 | No |
| 49 | PCDH9 |  |  | 1019 | -0.427 | 0.0315 | No |
| 50 | ROBO2 |  |  | 1037 | -0.438 | 0.0406 | No |
| 51 | ZBTB20 |  |  | 1061 | -0.451 | 0.0455 | No |
| 52 | CUX1 |  |  | 1071 | -0.455 | 0.0618 | No |
| 53 | DAAM1 |  |  | 1161 | -0.523 | 0.0183 | No |
| 54 | SRGAP3 |  |  | 1188 | -0.567 | 0.0268 | No |
| 55 | PDE1C |  |  | 1232 | -0.651 | 0.0262 | No |
| 56 | SYNE2 |  |  | 1282 | -0.829 | 0.0300 | No |
Table: GSEA details [plain text format]

  

Fig 2: RICHARDS\_NOWAKOWSKI\_SCIENCE\_2017\_IPC\_NEN1\_UPREG: Random ES distribution      
 Gene set null distribution of ES for **RICHARDS\_NOWAKOWSKI\_SCIENCE\_2017\_IPC\_NEN1\_UPREG**
